# Increased symplasmic permeability in barley root epidermal cells correlates with defects in root hair development

**DOI:** 10.1111/plb.12066

**Published:** 2013-08-08

**Authors:** M Marzec, A Muszynska, M Melzer, H Sas-Nowosielska, E U Kurczynska, S Wick

**Affiliations:** 1 Department of Genetics, Faculty of Biology and Environmental Protection, University of SilesiaKatowice, Poland; 2 Laboratory of Cell Biology, Faculty of Biology and Environmental Protection, University of SilesiaKatowice, Poland; 3 Department of Physiology and Cell Biology, Leibniz Institute of Plant Genetics and Crop Plant Research (IPK)Gatersleben, Germany; 4 Department of Plant Anatomy and Cytology, Faculty of Biology and Environmental Protection, University of SilesiaKatowice, Poland

**Keywords:** 8-Hydroxypyrene-1,3,6-trisulphonic acid, trisodium salt, cell differentiation, fluorescein diacetate, *Hordeum vulgare*, root hair cells, symplasmic communication

## Abstract

It is well known that the process of plant cell differentiation depends on the symplasmic isolation of cells. Before starting the differentiation programme, the individual cell or group of cells should restrict symplasmic communication with neighbouring cells. We tested the symplasmic communication between epidermal cells in the different root zones of parental barley plants *Hordeum vulgare* L., cv. ‘Karat’ with normal root hair development, and two root hairless mutants (*rhl1.a* and *rhl1.b*). The results clearly show that symplasmic communication was limited during root hair differentiation in the parental variety, whereas in both root hairless mutants epidermal cells were still symplasmically connected in the corresponding root zone. This paper is the first report on the role of symplasmic isolation in barley root cell differentiation, and additionally shows that a disturbance in the restriction of symplasmic communication is present in root hairless mutants.

## INTRODUCTION

Barley (*Hordeum vulgare* L.) is one of the major cereals cultivated worldwide. In a time where there is a rapid increase in human population and the absence of new areas under cultivation, improvement of cereals and other crops is a major task of plant research. Root systems play a critical role in plant growth and development, since they are responsible for water and nutrient uptake from the soil (Cailloux 1972; Itoh & Barber 1983; Föhse *et al*. 1991). A larger surface interface between the roots and the soil allows more efficient nutrient uptake by plants (Bates & Lynch 2000). One morphological strategy to increase root–soil (rhizosphere soil) contact is to develop root hairs, the tubular outgrowths of specialised root epidermal cells, called trichoblasts (Grabov & Bottger 1994; Lauter *et al.* 1996). It has been proved that barley cultivars with longer root hairs absorb more phosphorus from rhizosphere soil than cultivars with shorter root hair (Gahoonia *et al.* 2001). Moreover, an analysis of the root hairless mutant *brb* (*bald root barley*) and its parental variety ‘Pallas’ revealed almost twofold depletion of inorganic and organic phosphorus fractions compared to mutants without root hairs (Gahoonia *et al.* 2001).

Although root hairs play an important role in the uptake of nutrients and the production of biomass by crops, their development in monocotyledons is still not well understood. However, a few genes involved in root hair differentiation in monocotyledons have been described recently, *e.g*. β-expansin in barley (*HvEXPB1*; Kwaśniewski & Szarejko 2006), apyrase (*OsAPY1*; Yuo *et al.* 2009) and cellulose synthase-like D1 (*OsCSLD1*; Kim *et al.* 2007) in the root hairs of rice. However, in comparison to *Arabidopsis thaliana,* where a dozen or so genes have been recognised for each stage of root hair development (for review see Horn *et al.* 2009), our knowledge on the genetic control of root hair development in crop plants is limited. Lately identified root hair mutants of crop plants (Wen & Schnable 1994; Engvild & Rasmussen 2004; Szarejko *et al.* 2005; Rebouillat *et al.* 2009) are very useful for gene identification and recognition of molecular mechanisms underlying the process of differentiation of root epidermal cells in monocots.

Many studies have demonstrated that the process of plant cell differentiation depends on symplasmic isolation of these cells (Rinne & van der Schoot 1998; Ruan *et al.* 2004; Kim & Zambryski 2005; Kurczyńska *et al*. 2007). The process of symplasmic communication depends on the presence of plasmodesmata – the cytoplasmic channels within cell walls of neighbouring cells (Lucas *et al.* 1993; Roberts & Oparka 2003). Plasmodesmata are structures that regulate the passage of molecules between plant cells (Crawford & Zambryski 2001; Maule 2008) that constitute the route between neighbouring cells for proteins and RNAs (Oparka & Cruz 2000; Lucas *et al.* 2001; Jorgensen 2002; Ruiz-Medrano *et al.* 2004). It has been postulated that only when plasmodesmata are closed or absent can cells realise their own individual genetic programme. Symplasmic isolation is involved in development of the embryo sac in *Torenia fournieri* (Han *et al.* 2000; Dresselhaus 2006), establishment of the apical–basal axis and tissue differentiation during embryogenesis in *Arabidopsis* (Kim & Zambryski 2005; Kim *et al.* 2005; Stadler *et al.* 2005), androgenesis in barley (Wrobel *et al.* 2011) and the differentiation of root epidermal cells in *Arabidopsis* (Duckett *et al.* 1994). Duckett *et al*. (1994) showed that in the meristematic zone of the *Arabidopsis* root all epidermal cells were symplasmically connected, whereas in the differentiation zone, both types of epidermal cell, trichoblasts and atrichoblasts, were symplasmically isolated from neighbouring cells. These results confirmed the key role of symplasmic isolation in plant cell differentiation, especially in initially homogeneous cell systems. The explanation of why symplasmic isolation is so important for the differentiation of root epidermal cells may be the fact that some proteins involved in root hair development in *Arabidopsis* may be transported *via* plasmodesmata. The *CPC* (*CAPRICE*) gene, a positive regulator of root hair formation, is transcribed only in atrichoblasts, but CPC protein is present in all root epidermal cells (Wada *et al.* 2002; Kurara *et al.* 2005). On the other hand, negative regulators of root hair formation, such as GL3 (GLABRA3) and EGL3 (ENHANCER OF GLABRA3), may be transported through plasmodesmata from trichoblasts to atrichoblasts (Bernhardt *et al.* 2005). CPL3 (CAPRICE-LIKE MYB3) is another protein that is also involved in root hair development and is transported *via* plasmodesmata (Tominaga *et al.* 2008). On the basis of results obtained to date, new models depicting the differentiation of root epidermal cells in *Arabidopsis*, which critically depend on the movement of proteins between neighbouring cells *via* plasmodesmata, have been proposed (Benítez *et al*. 2008; Petricka & Benfey 2008; Savage *et al.* 2008). Furthermore, a similar dependence between cell differentiation and the movement of proteins through plasmodesmata was observed in the leaf epidermis of *Arabidopsis* (Digiuni *et al.* 2008; Zhao *et al.* 2008). These results show that genetic control of cell fate and patterning may be supported by the restriction of symplasmic communication.

The main aim of the presented studies was to describe symplasmic communication between different cells of the barley root epidermis during cell differentiation in the parental variety and two root hairless mutants, *rhl1.a* and *rhl1.b*, in order to understand whether there is any difference in the transport *via* plasmodesmata between the analysed genotypes. This would suggest the importance of symplasmic isolation during the differentiation of barley root hairs.

## MATERIAL AND METHODS

### Plant material

The mutants analysed were obtained after treatment with *N-*methyl-*N*-nitrosourea (MNU) and sodium azide (NaH_3_) of spring barley (*Hordeum vulgare* L., cv. ‘Karat’) at the Department of Genetics, University of Silesia. Both mutants (*rhl1.a* and *rhl1.b*) are completely hairless and their root hair phenotypes are controlled *via* a single recessive gene (Szarejko *et al.* 2005).

The seeds of barley mutants were surface sterilised with a 20% bleach solution (v/v) and germinated in hydroponic conditions using glass tubes sealed with Parafilm. All of the plants investigated were grown in a growth chamber under a 16-/8-h photoperiod at 20 °C, 180 μmol·m^−2^·s^−1^ light intensity. All experiments were carried out on 5-day-old seedlings.

### Light microscopy

For histological examination, combined conventional and microwave-assisted fixation, substitution and embedding of 2-mm root segments were performed using a PELCO BioWave 34700-230 (TedPella, Redding, CA, USA) according to the procedure described in Thiel *et al*. (2012). The semi-thin sections (*ca*. 2-μm thick) were cut from the embedded samples, mounted on slides and stained for 2 min with 1% (w/v) methylene blue/1% (w/v) Azur II in 1% (w/v) aqueous borax at 60 °C prior to light microscopic examination with a Zeiss Axiovert 135 microscope.

Transverse and longitudinal sections of at least 20 roots from different individuals of ‘Karat’ and both mutants were studied at each root developmental zone: meristematic, elongation, differentiation and mature root hair zone (3 cm from the tip).

### Fluorescence microscopy analysis of symplasmic transport tracers in roots

#### Fluorescein diacetate (FDA)

FDA (Sigma-Aldrich, Cat. No. F7378, Poznan, Poland) stock solution prepared in acetone (5 mg·ml^−1^) was dissolved in demineralised water (0.5 ml per 24.5 ml) to obtain FDA working solution. Roots, still attached to the whole plant, were treated with the FDA working solution in darkness for 15 min. The roots were then washed in demineralised water, placed on microscopic slides and covered with a cover glass.

Fluorescence recovery after photobleaching (FRAP) was used to monitor movement of the FDA within the epidermis of the barley root. For fluorescence analyses, a 20 ×  PlanFluor objective lenses (NA 0.5) and a scan format of 512 × 512 pixels was used in an Olympus FV1000 confocal system (Olympus, Warszawa, Poland). The pre- and post-bleaching events were imaged using 0.5% power of a 488-nm line from an argon-ion laser (Melles Griot BV, the Netherlands); emission was detected using a 505–530 nm band pass filter. For photobleaching of the fluorescence in a single cell, 90% power of the laser line was used for 10 s. This procedure was repeated at least ten times for each root zone of all genotypes.

#### 8-Hydroxypyrene-1,3,6-trisulphonic acid, trisodium salt (HPTS)

In order to monitor the movement of the HPTS (5 mg·ml^−1^ in demineralised water; Sigma-Aldrich, Cat. No. H1529) in barley root cells, the tracer was injected into cortical cells of the meristematic zone using a tissue cell pressure probe (Steudle 1993) provided with a microcapillary preceded by treatment with a 0.1 mM demineralised water solution of DDG (2-deoxy-d-glucose; Sigma-Aldrich, Cat. No. D4601) for 30 min to inhibit deposition of callose during injection. Observations of roots after the injection were carried out under an Olympus BX40 epifluorescence microscope equipped with an Olympus 3040 CCD camera (Olympus). Tracer was excited with 480-nm light produced by a UV lamp, and a 500–530 nm band pass filter was used for excitation of HPTS (Mercury BX-FLA fluorescence illuminator, Olympus, Warszawa, Poland). Injection analyses were carried out on ten roots from each genotype.

### Electron microscopy analysis

#### Ultrastructure analysis

For ultrastructure examination, combined conventional and microwave-assisted fixation, substitution and embedding of 2-mm root segments was performed using a PELCO BioWave 34700-230 (TedPella) according to the procedure described in Thiel *et al*. (2012). For electron microscopy analysis with a Tecnai Sphera G2 (FEI Co., Eindhoven, the Netherlands) transmission electron microscope at 120 kV, ultrathin sections *ca*. 70-nm thick were cut with a diamond knife and contrasted with a saturated methanolic solution of uranyl acetate and lead citrate before examination. For each genotype, sections from at least ten roots were analysed.

#### Immunogold analysis

Roots used for immunogold labelling of callose were cut into 2-mm segments, vacuum infiltrated for a short period with 50 mM cacodylate buffer (pH 7.2) containing 0.5% (v/v) glutaraldehyde and 2.0% (v/v) formaldehyde, and kept for 4 h at room temperature in the same medium. Samples were washed with the same buffer for 15 min, followed by two washing steps for 15 min with distilled water. Dehydration of samples was done step-wise, increasing the concentration of ethanol. The steps were performed as follows: 30% (v/v), 40% (v/v), 50% (v/v), 60% (v/v), 70% (v/v), 90% (v/v) and two times 100% (v/v) ethanol for 45 min each. After dehydration the samples were infiltrated subsequently with Lowycryl HM20 resin (Plano, Wetzlar, Germany): 25% (v/v) HM20 resin in ethanol overnight, 50% (v/v) and 75% (v/v) for 4 h each and then 100% HM20 overnight. Samples were transferred into gelatin capsules and an automated freeze substitution unit (Leica Microsystems, Bensheim, Germany), kept there for 3 h in fresh resin at −35 °C and polymerised at −35 °C for 72 h in UV light. Immunogold labelling of 70-nm ultrathin sections was carried out as described previously (Teige *et al.* 1998). Anti-mouse monoclonal (1,3)-b-D-glucan antibody (Biosupplies, Parkville, Vic., Australia) 1:100 diluted was used as a primary antibody against callose, and goat anti-mouse conjugated to 10-nm gold particles was used as a secondary antibody. The ultrathin sections were contrasted in LEICA EM stain (Leica Microsystems) with uranyl acetate (Polyscience Inc., Eppelheim, Germany) for 30 min and subjected to transmission electron microscope (Tecnai Sphera G2) at 120 kV. Sections from at least seven roots from each genotype were analysed.

## RESULTS

**Figure 1:**
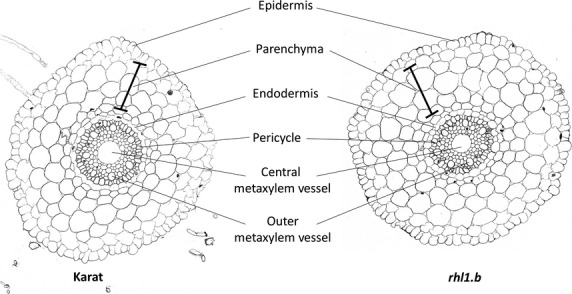
Histology of roots of the mutant *rhl1.b* and parental variety ‘Karat’ of *Hordeum vulgare* L. Cross-sections through the root differentiation zone.

Considering their morphology and histology, the phenotype of ‘Karat’ roots was similar to those of barley described earlier (Clowes 2000). Three zones were detected along the root longitudinal axis: meristematic, elongation and differentiation zones. Histological analysis of roots of both the parental variety and the mutants showed the presence of: epidermis, parenchyma, endodermis, pericycle and central root stele (Fig.1). For comparison of the root anatomy of the parental variety and the two mutants, sections from 20 different roots of 20 different plants for each genotype were analysed. There were no differences between parental plants and mutants in the case of cell size, number of epidermal cells in traverse sections, number of parenchyma cell layers and number of metaxylem vessels (Fig.1).

**Figure 2:**
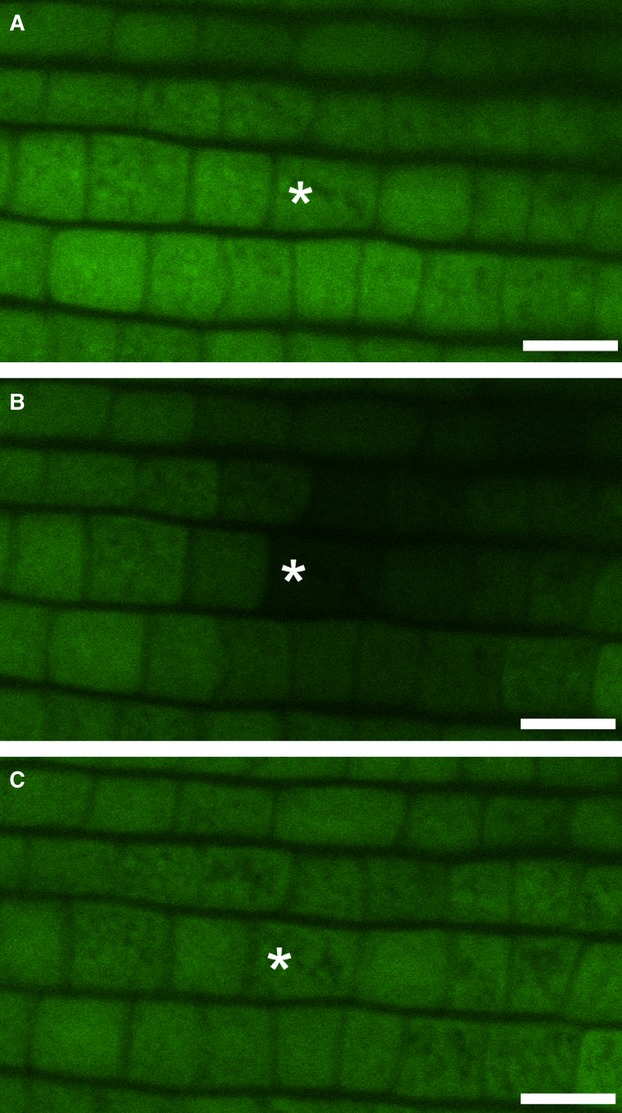
Fluorescence analysis of the symplasmic tracer FDA in epidermal root cells during the FRAP experiment. The meristematic zone of ‘Karat’ before photobleaching (A): immediately after photobleaching (B): and 30 min after photobleaching (C): Bar = 20 μm.

**Figure 3:**
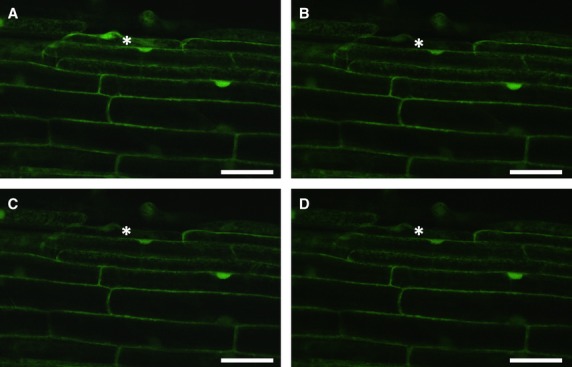
Lack of symplasmic communication between root epidermal cells of ‘Karat’. The differentiation zone of ‘Karat’ before photobleaching (A): immediately after photobleaching (B): 10 min after photobleaching (C): and 30 min after photobleaching (D): Bar = 50 μm.

**Figure 4:**
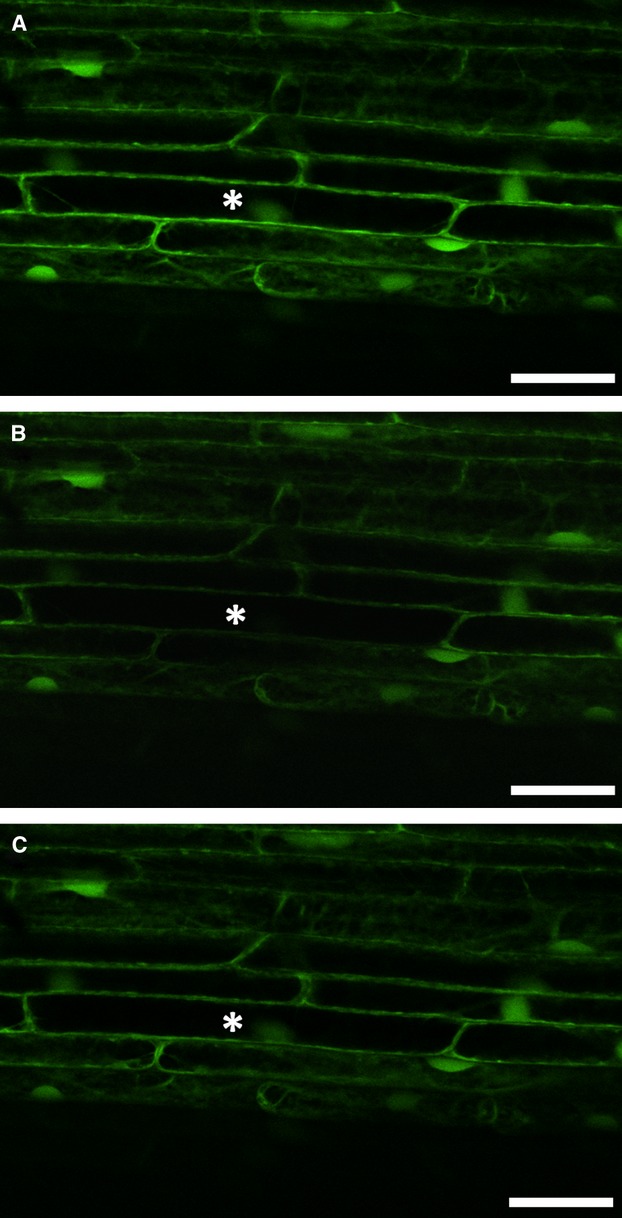
Symplasmic communication between root epidermal cells of *rhl1.b*. The differentiation zone of the mutant before photobleaching (A): immediately after photobleaching (B): and 10 min after photobleaching (C): Bar = 50 μm.

Symplasmic communication between epidermal root cells was monitored using a confocal laser scanning microscope and FRAP (Fig.2). In the meristematic zone of all three genotypes (‘Karat’, *rhl1.a* and *rhl1.b*), recovery time of fluorescence was very short: 0.25 min in *rhl1.a* and 0.5 min in other genotypes analysed (Table1). In the apical elongation zone, the fluorescence recovery time was 3 min, regardless of root genotype. The most visible differences were observed in the basal part of the elongation and differentiation zones. In the basal part of the elongation zone, the shortest observed time was in the *rhl1.a* mutant, which was more than three times shorter in comparison to ‘Karat’ and the *rhl1.b* mutant. No recovery of fluorescence was observed in the differentiation zone of ‘Karat’, regardless of which cell (producing or not producing root hairs) was photobleached (Fig.3, Table1), while in both mutants the time needed for recovery was about 10 min within the differentiation zone (Fig.4, Table1). The obtained results suggest that in both mutants all epidermal cells in the differentiation zone were symplasmically connected, whereas in the parental variety, root hair cells and non-root hair cells were symplasmically isolated from neighbouring cells (Table1). These results were also confirmed using caged fluorescein (data not shown).

**Table 1:** Transport of fluorescent probes between root epidermal cells. Quantification of fluorochrome movement after photobleaching of *Hordeum vulgare* L., cv. ‘Karat’ root epidermal cells at different stages of development

	‘Karat’				
	mz	aez	bez	dz	
				hc	nhc
% bleached cells that recovered fluorescence (n)	100 (15)	100 (10)	53.8 (13)	8.3 (12)	14.3 (14)
	0.5 min	3.0 min	10.0 min	nfr	
Average time of fluorescence recovery after photobleaching	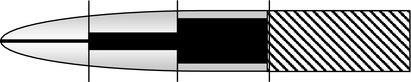				

	*rhl1.a*				
	mz	aez	bez	dz
% bleached cells that recovered fluorescence (n)	100 (10)	100 (12)	92.9 (14)	78.6 (14)
Average time of fluorescence recovery after photobleaching	0.25 min	3.00 min	3.00 min	10.00 min
	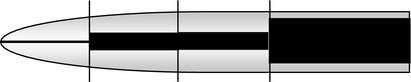			

	*rhl1.b*				
	mz	aez	bez	dz
% bleached cells that recovered fluorescence (n)	100 (10)	100 (14)	84.6 (13)	84.6 (13)
Average time of fluorescence recovery after photobleaching	0.5 min	3.0 min	10.0 min	10.0 min
	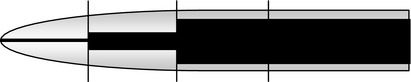			

The width of the black rectangle schematically represents the time of fluorescence recovery, the striped rectangle represents no fluorescence recovery; aez, apical elongation zone; bez, basal elongation zone; dz, differentiation zone; hc, hair cell; mz, meristematic zone; n, total number of analysed cells; nhc, non-hair cell; nfr, no fluorescence recovery.

**Figure 5:**
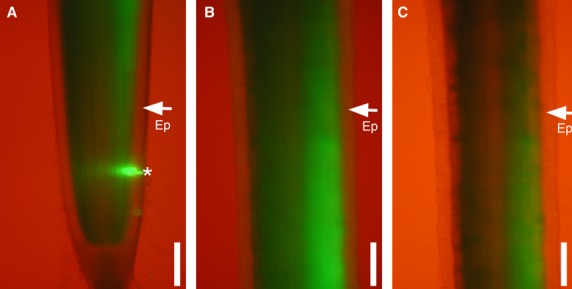
Fluorescence analysis of the HTPS symplasmic tracer in roots of *rhl1.a* after injection into the cortex of the meristematic zone. Meristematic zone (A): elongation zone (B): and differentiation zone (C): A star indicates the position of fluorochrome injection; arrows indicate the epidermis (Ep). Bar = 100 μm.

The next question was whether epidermal cells are symplasmically connected to other root tissues in different zones of barley root. In order to investigate this, HPTS was inserted with a cell pressure probe. After local insertion into cortical cells, it was clearly visible that the fluorochrome did not pass to epidermal cells but freely shifted to apical and basal part of the root within the other cortical cells. These results were obtained for ‘Karat’ and both allelic root hairless mutants (Fig.5).

**Figure 6:**
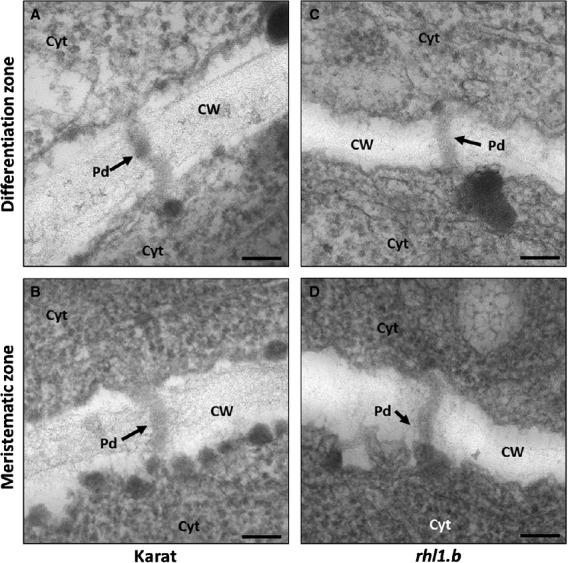
The ultrastructure of plasmodesmata. No differences were observed in the structure of plasmodesmata localised between root epidermal cells of the ‘Karat’ differentiation zone (A): and meristematic zone (B): Plasmodesmata in differentiation zone (C): and meristematic zone (D): of *rhl1.b* showed no differences. Pd, Plasmodesmata, Cyt, cytoplasm, CW, cell wall, bar = 100 μm.

To understand symplasmic isolation during differentiation of barley epidermal cells, the ultrastructure of plasmodesmata and callose deposition in the parental variety and the *rhl1.b* mutant was analysed. No differences in plasmodesmata ultrastructure between plasmodesmata connecting the neighbouring epidermal cells in the meristematic zone, zone of cell elongation and zone of cell differentiation in ‘Karat’ were observed. In the case of the wild type, only simple plasmodesmata were observed in the root epidermis and not a single branched plasmodesmata was found in any of analysed zones of the root (Fig.6A,B). Similar results were obtained for *rhl1.b*; there were no differences between plasmodesmata localised in different zones of roots (Fig.6C,D), nor between the parental variety and mutant (Fig.6).

**Figure 7:**
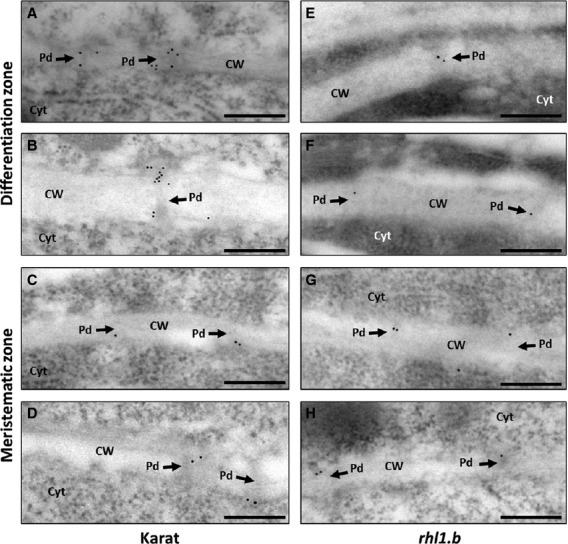
Immunolocalisation of callose in plasmodesmata. Callose deposition in plasmodesmata labelled with 10-nm gold particles in the periclinal cell wall of two epidermal cells of ‘Karat’ roots at the differentiation zone (A, B): and meristematic zone (C, D): and of the *rhl1.b* mutant root differentiation zone (E, F): and meristematic zone (G, H): Pd, Plasmodesmata, Cyt, cytoplasm, CW, cell wall, bar = 200 μm.

Immunogold localisation of callose in ‘Karat’ revealed increasing deposition of this polysaccharide inside plasmodesmata between epidermal cells during their development. In the meristematic zone of roots, single molecules of callose had gold-labelled antibodies (Fig.7A,B); whereas in the zone where root epidermal cells started elongation and differentiation, a higher number of callose molecules was observed (Fig.7C,D). In contrast, in the case of the *rhl1.b* mutant, comparable amount of callose was deposited in plasmodesmata between root epidermal cells, regardless of the zone of the root (Fig.7E–H).

## DISCUSSION

It is well known that cell-to-cell communication *via* plasmodesmata is important in determining the direction of cell differentiation (Rinne & van der Schoot 1998; Ruan *et al.* 2004; Kim & Zambryski 2005; Kurczyńska *et al*. 2007). The presented results show that in the ‘Karat’ variety during normal development of root epidermal cells into trichoblasts and atrichoblasts, symplasmic communication between epidermal cells decreased during cell differentiation. Communication *via* plasmodesmata between epidermal cells was shown in the meristematic zone of all three genotypes analysed; similar results were obtained for the same root part in *A*. *thaliana* (Duckett *et al*. 1994). Symplasmic transport between all epidermal cells indicates that at this stage of development the epidermis is a single symplasmic domain connected by open plasmodesmata; as also found for undifferentiated group of cells such as young embryo sacs of *Torenia fournieri* before fertilisation (Han *et al.* 2000) or young embryos of *Arabidopsis* (Kim & Zambryski 2008). In all of these cases, neighbouring epidermal cells were connected *via* open plasmodesmata. The results presented in this paper show that reduced symplasmic communication between trichoblasts and atrichoblasts occurs in the elongation zone of roots of the parent variety. Additionally, complete symplasmic isolation was detected in the mature root hair zone of the ‘Karat’ variety, what indicates that the root epidermis is no longer a single symplasmic domain but that symplasmic subdomains are generated within this tissue, including domains built by individual atrichoblasts and individual trichoblasts. Moreover, in both of the analysed root hairless mutants the restriction of symplasmic communication between root epidermal cells during cell elongation and differentiation was lower. It is well documented that plasmodesmata are the route for molecules such as RNAs and proteins, which play a key role during plant cell differentiation (Zambryski 2012). This is why symplasmic isolation of plant cells is necessary in order to start the individual developmental programme of a single or a group of cells. This could be why increased symplasmic permeability in barley root epidermal cells correlates with defects in root hair development. In addition, the obtained results suggest that symplasmic isolation or restriction of symplasmic communication is essential for the correct development of root hairs in barley, as in the case of *Arabidopsis* (Duckett *et al.* 1994). It is well documented that the number of different symplasmic domains present in a plant organ is positively correlated with the stage of cell development – in the mature stage of plant development there are a higher number of symplasmic domains present (Stadler *et al.* 2005), as found here for the mature root hair zone of barley.

In the presented paper HPTS and FDA were used as tracers with a molecular diameter around 0.9 nm (Heinlein & Epel 2004). In this case, the term ‘symplasmic isolation’ means that restriction of the plasmodesmata pathway concerns molecules with a diameter of 0.9 nm and more, suggesting that transport of water (0.96-nm molecular diameter) and nutrients *via* symplasm may occur without hindrance.

The presented results show that symplasmic communication between root epidermal cells in the two analysed root hairless mutants differed from the parent variety. In both mutants, movement of symplasmic tracers between epidermal cells was detected in all investigated root zones, *i.e*. the differentiation of cells is correlated with the degree of symplasmic communication between them. This is a first report indicating that in root hairless mutants, root epidermal cells are still symplasmically connected in all root zones, whereas symplasmic communication is restricted in wild-type plants. Moreover, this is the first time that a disturbance in regulation of symplasmic communication could be correlated with the root hairless phenotype in plants. Symplasmic isolation may take place in two different ways: by decreasing the number of plasmodesmata between cells, as in the case of guard cells (Wille & Lucas 1984), or by blocking the plasmodesmata with callose, as in *Chara vulgaris,* L. during spermatogenesis (Kwiatkowska & Maszewski 1985). Immunogold localisation of callose indicates that higher deposition of this polysaccharide plays a crucial role in the restriction of symplasmic communication between barley root epidermal cells during their differentiation. Moreover, the lack of increasing deposition of callose inside the plasmodesmata in the mutant with a root hairless phenotype confirms our hypothesis on the importance of symplasmic isolation in the process of cell differentiation. Although, also in both analysed mutants, there was a longer fluorescence recovery time in the elongation and differentiation zones in comparison to the meristematic zone of the root. These results may be explained by the lower number of plasmodesmata between cells localised above the meristematic zone; however this hypothesis remains to be confirmed during further experiments. The future work will focus on analysis of the number and distribution of plasmodesmata in barley epidermal cells of the root epidermis during differentiation, and also on analysis of expression profiles of genes encoding barley callose synthase proteins in roots of the parent varieties and different mutants of barley.

## CONCLUSION

Our studies show that symplasmic communication between trichoblasts and atrichoblasts decreases gradually during the differentiation of these two types of cell in the parental variety of barley. This means that the mechanism of specialisation of epidermal root cell in monocots is similar to that of dicots. In root hairless mutants such a decrease in symplasmic communication was not observed, and this is the first report describing disorders in the regulation of symplasmic communication in root hairless mutants. To identify any correlations between the phenotype and the regulation of symplasmic communication in roots, additional studies are required. Root hairless mutants provide a good subject for future research, not only in terms of root hair development but also for symplasmic communication between cells.
